# New diffusion metrics for spondylotic myelopathy at an early clinical stage

**DOI:** 10.1007/s00330-012-2410-9

**Published:** 2012-03-13

**Authors:** Masaaki Hori, Issei Fukunaga, Yoshitaka Masutani, Atsushi Nakanishi, Keigo Shimoji, Koji Kamagata, Koichi Asahi, Nozomi Hamasaki, Yuriko Suzuki, Shigeki Aoki

**Affiliations:** 1 Department of Radiology, School of Medicine, Juntendo University, 2-1-1 Hongo, Bunkyo-ku, Tokyo 113-8421 Japan; 2 Department of Health Science, Graduate School of Human Health Sciences, Tokyo Metropolitan University, 7-2-10, Higashiogu, Arakawa, Tokyo 116-8551 Japan; 3 Division of Radiology and Biomedical Engineering, Graduate School of Medicine, The University of Tokyo, 7-3-1 Hongo, Bunkyo-ku, Tokyo 113-8655 Japan; 4 Philips Electronics Japan, Ltd, Philips Bldg. 13-37 Kohnan 2-chome, Minato-ku, Tokyo 108-8570 Japan

**Keywords:** Cervical spondylosis, Spinal cord, Non-Gaussian, Diffusion kurtosis imaging, Diffusion tensor imaging

## Abstract

**Objectives:**

To investigate the use of root mean square displacement (RMSD) and mean diffusional kurtosis (DK) metrics of *q*-space imaging data to estimate spinal cord compression in patients with early cervical spondylosis.

**Methods:**

We studied 50 consecutive patients at our institution (22 male, 28 female; mean age 58 years; age range 20–86 years) who had clinical signs and symptoms suggestive of early clinical stage cervical myelopathy. After conventional magnetic resonance (MR) imaging, diffusion tensor and *q*-space image data were acquired using 3-T MR imaging. Fractional anisotropy (FA), apparent diffusion coefficient (ADC), RMSD and mean DK values were calculated and compared between compressed and uncompressed spinal cords.

**Results:**

FA and mean DK values were significantly lower and RMSD was significantly higher (*P* = 0.0060, 0.0020 and 0.0062, respectively; Mann–Whitney *U* test with the Bonferroni correction) in compressed spinal cords than in uncompressed cords. ADC was also higher in compressed cords, but this difference was not statistically significant.

**Conclusions:**

In the evaluation of spinal cord damage in early cervical spondylosis, mean DK and RMSD values in the spinal cord may be highly sensitive indicators of microstructural change and damage.

**Key Points:**

• *Absolute surgical indications for cervical spondylosis with myelopathy remain to be established*.

• *Diffusion tensor MRI shows abnormalities in normal-appearing but compressed spinal cord*.

• *Non-Gaussian diffusion analysis is highly sensitive in revealing spinal cord damage*.

## Introduction

Cervical spondylosis is a common degenerative disease. In this condition, disk degeneration, bone formation with osteophytic changes, and hypertrophy of the ligamentum flavum can result in spinal canal narrowing and cervical cord myelopathy. Routine clinical magnetic resonance (MR) imaging is widely performed to evaluate morphological changes in cervical spondylosis, but there is only a weak correlation between MR imaging findings and clinical symptoms [1]. Particularly in the early clinical stages, no abnormal intensity is observed in the spinal cord on conventional MR images, despite the clinical manifestations. In fact, absolute surgical indications for cervical spondylosis with myelopathy remain to be established [2]. Therefore, in the clinical situation, an objective method of using MR imaging to estimate damage in patients with cervical spondylosis is desirable.

In addition to conventional morphologic MR imaging, diffusion tensor imaging (DTI) has been proposed for evaluating microstructural changes in the brain. Signal changes in DTI are based on the diffusion of water molecules in tissues, and characteristic quantitative values, such as the fractional anisotropy (FA) and apparent diffusion coefficient (ADC), can be calculated [3]. In short, the FA value is used most frequently to measure diffusion anisotropy, and the ADC is a scalar value that reflects molecular diffusivity when motion is restricted, for example by fluid viscosity. DTI can be used with higher sensitivity and specificity than conventional MR imaging to estimate white matter changes in neurodegenerative disease [4] or demyelinated lesions [5]. In the spinal cord, DTI with FA or ADC measurements shows promise for detecting and quantifying the pathology of spinal cord abnormalities. Reduced FA and increased ADC have been observed in the spinal cord at compressed sites and are presumably explained by microstructural changes [6, 7]. Recently, a more advanced form of diffusion analysis, *q*-space imaging (QSI) analysis, has emerged. This technique does not require the assumption of a Gaussian shape and model of water molecules. It has shown promise for evaluating brain and spinal disorders in vivo [8–13] because it can provide additional and different diffusion metrics, namely the root mean square displacement (RMSD) and diffusional kurtosis (DK) [14–20], which give in vivo microstructural information that complements the ADC and FA values. For example, increased ADC can indicate either decreased viscosity of the tissue or spatial dilatation of the water movement space [21]. It is difficult to distinguish between these phenomena by using ADC values only. However, the RMSD values reflect the real extent of water molecule movement. We therefore hypothesized that QSI analysis would be able to provide more information on structural and pathological changes in the spinal cord in vivo.

Our aim was to investigate the use of RMSD and mean DK metrics derived from QSI data to estimate spinal cord compression in patients with early cervical spondylosis.

## Materials and methods

### Subjects

Between 28 October 2010 and 25 February 2011, a total of 50 consecutive patients at our institution (22 male, 28 female; mean age 58 years; age range 20–86 years) with clinical signs and symptoms suggestive of early cervical myelopathy participated in this study. Informed consent was obtained from each patient. We obtained ethics approval from the institutional review board before the study. Exclusion criteria were as follows: (a) the presence of other intraspinal disease; (b) the presence of abnormal intensity in the spinal cord on conventional MR imaging (T1- or T2-weighted imaging); (c) a history of surgery to the neck for any disease; and (d) unsatisfactory image quality for calculating diffusion metrics.

### Image acquisition

All images were acquired by using 3-T MR (Achieva; Philips Medical Systems, Best, Netherlands). After turbo spin-echo T1- and T2-weighted sagittal and axial imaging, diffusion tensor and *q*-space image data were acquired. Imaging parameters for sagittal images were as follows: repetition time (ms)/echo time (ms) 612.5/7.7 for T1-weighted imaging (T1WI) and 3,000/90 for T2-weighted imaging (T2WI); echo train length 4 for T1WI and 21 for T2WI; number of signals acquired 2; section thickness/gap 4/0.4 mm; 12 sections; field of view 220 × 220 mm; matrix 512 × 512. Imaging parameters for axial images were as follows: repetition time (ms)/echo time (ms) 606.9/9.9 for T1WI and 4300/90 for T2WI; echo train length 5 for T1WI and 17 for T2WI; number of signals acquired 2; section thickness/gap 5/0.5 mm; 20 sections; field of view 160 × 160 mm; matrix 512 × 512. Parameters used for the diffusion tensor imaging and QSI were as follows: repetition time (ms)/echo time (ms) 6,996/73; number of signals acquired 1; section thickness/gap 3/0 mm; 30 sections; field of view 80 × 80 mm; matrix 64 × 64 (128 × 128 reconstructed); imaging time approximately 7 min; and six *b* values (0, 400, 800, 1,200, 1,600 and 2,000 s/mm^2^) with diffusion encoding in 6 directions for every *b* value. Corresponding *q* values for each *b* value were 175.9, 248.8, 304.7, 351.9 and 393.4 cm^–1^, respectively. Gradient length (δ) and time between the two leading edges of the diffusion gradient (Δ) were 10.7 and 36.3 ms, respectively.

### Diffusion tensor, *q*-space and kurtosis imaging analysis of the spinal cord

Diffusion tensor, *q*-space and kurtosis analyses were performed by using the free software dTV II FZR and Volume-One 1.72 (Image Computing and Analysis Laboratory, Department of Radiology, The University of Tokyo Hospital, Tokyo, Japan) [7] on an independent Windows PC.

**Fig. 1:**
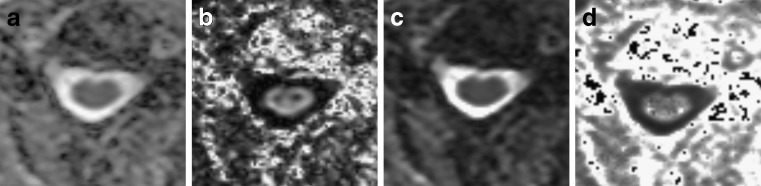
Diffusion metric maps of the cervical spinal cord in the axial plane. Apparent diffusion coefficient (**a**), fractional anisotropy (**b**), full width at half maximum of probability density function (**c**) and mean diffusional kurtosis (**d**) at the C3–C4 vertebral level

First, FA and ADC maps, based on the conventional mono-exponential model, were calculated (Fig. 1). Because the *q*-space image data included multiple *b* value data, FA and ADC could be calculated by using part of the *q*-space data.

Next, the full width at half maximum (FWHM) of the probability density function (PDF) and mean DK maps were obtained. Details of the new diffusion metrics and their calculation procedure were as previously described [8–19]. Briefly, the key principle in *q*-space analysis is that a Fourier transform of the signal attenuation with respect to *q* (or the *b* value) provides the PDF for diffusion by using multiple *q* values [10]. The shape of the computed PDF can be characterized by the FWHM and the maximum height of the curve. In the specific case of unrestricted Gaussian diffusion, the diffusion constant *D* and the RMSD for one-dimensional diffusion can be computed from the FWHM. Mean RMSD was calculated from the FWHM values (RMSD = 0.425 × FWHM) [10, 11].

Moreover, as described in previous papers [14, 22], the DK for a single direction can be determined by acquiring data at three or more *b* values (including *b* = 0) and fitting them to Eq. (1):

1 $$ {\text{ln}}[{\text{S}}({\text{b}})] = {\text{ln}}[{\text{S}}(0)]-{\text{b}} \cdot {{\text{D}}_{{{\text{app}}}}} + {\text{1}}/{\text{6}} \cdot {{\text{b}}^{{\text{2}}}} \times {\text{D}}_{{{\text{app}}}}^{2} \times {{\text{K}}_{{{\text{app}}}}} $$

where *D*_app_ is the apparent diffusion coefficient for the given direction and *K*_app_ is the apparent kurtosis coefficient, which is dimensionless.

**Fig. 2:**
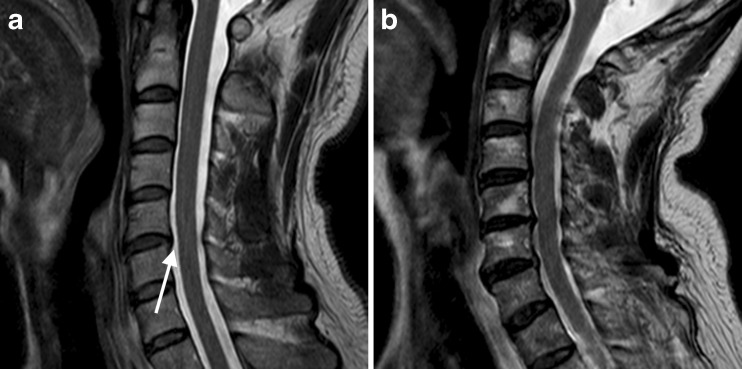
T2-weighted fast spin-echo images in the sagittal plane of one patient from group 1 (**a**) and one patient from group 2 (**b**). High intensity indicates cerebrospinal fluid between the cervical spinal cord and dura in **a** (*arrow*); this is absent in the compressed areas in **b**

By referring to T2-weighted images, two experienced neuroradiologists (M.H. and S.A.) classified the patients into two groups in consensus: (1) uncompressed spinal cords (residual cerebrospinal fluid (CSF) signal between the spinal column and spinal cord on the T2-weighted images); and (2) compressed spinal cords (cervical canal narrowing and no residual CSF signal between the spinal column and spinal cord at the level of narrowing on T2-weighted images) (Fig. 2). On the axial images of the calculated maps, a region of interest (ROI) was drawn manually on the spinal cord by an experienced neuroradiologist M.H.; the ROI included both white matter and grey matter, excluding any CSF contribution, at spinal canal levels C3–C4, C4–C5 and C5–C6. Therefore, the shape of each ROI varied according to the shape of the spinal cord. The dTV II FZR software allowed for copying of the ROIs and guaranteed the evaluation of the same region with diffusion metric maps. FA, ADC, RMSD and mean DK values were measured in each area. Areas with severe signal loss or calculation errors were excluded from the analysis.

### Statistical analysis

A preliminary analysis was performed by using the Anderson–Darling test to evaluate whether the data were normally distributed. Then, statistical comparisons between the two groups were performed by using IBM SPSS Statistics software (version 19.0; SPSS, Chicago, IL). A *P* value less than 0.05 was considered to indicate a statistically significant difference.

## Results

Thirty-three patients were included in the evaluation; 17 patients were excluded on the basis of the exclusion criteria described above. The excluded patients consisted of 15 patients whose diffusion images were degraded because of magnetic susceptibility and motion artefacts, one patient with spinal canal meningioma and one patient with syringohydromyelia.

There were 15 patients in group 1 (“uncompressed spinal cords”) and 18 patients in group 2 (“compressed spinal cords”). Their demographic characteristics are summarized in Table 1. Uncompressed spinal cords were measured in a total of 26 ROIs in the group 1 patients (11 ROIs at C3–C4 spinal canal level, 13 ROIs at C4–C5 and 2 ROIs at C5–C6); values (means ± SD) for FA, ADC (10^–3^ mm^2^/s), RMSD (μm) and mean DK were 0.656 ± 0.041, 0.939 ± 0.033, 8.287 ± 0.098 and 0.905 ± 0.095, respectively. Compressed spinal cords were measured in a total of 20 ROIs in the group 2 patients. Six ROIs were placed at the C3–4 spinal canal level, 9 ROIs at C4–5 and 5 ROIs at C5–6. Because the Anderson–Darling test had revealed that the data were not normally distributed, statistical comparison of groups was performed by using the Mann–Whitney *U* test with Bonferroni correction. Gender and age of patients were not considered as covariates for analysis, because a preliminary analysis using Spearman’s correlation showed no significant correlations between them and the diffusion metrics. The average size (mean ± SD) of the ROI (in pixels) was 58 ± 16 in group 1 and 57 ± 14 in group 2; no significant difference was seen between the groups in relation to ROI size. In the compressed spinal cords, FA (0.606 ± 0.075) and mean DK (0.802 ± 0.095) were significantly lower and RMSD (8.446 ± 0.269) was significantly higher (*P* = 0.0060, 0.0020 and 0.0062, respectively) than in the uncompressed cords. Increased ADC (0.977 ± 0.134) was also observed in the compressed cords, but this difference was not significant (*P* = 0.45). Although symptoms of numbness and pain tended to be more frequently observed in group 2 than in group 1, statistical analysis was not able to be conducted because of the small number of patients.

**Table 1:** Demographic characteristics of subjects

	Group 1 (*n* = 15)	Group 2 (*n* = 18)
Sex (male/female)	7:8	6:12
Mean age (SD), years	50.5 (16.2)	63.3 (10.8)
Symptoms^a^		
Numbness	6	11
Pain	6	11
Cervical vertigo	2	1
Neck stiffness	2	2
Hypalgesia	2	1
Tremor	1	2
Apraxia	1	1
Jitteriness	1	0

^a^Multiple symptoms were reported by some patients

## Discussion

This is the first evaluation of various diffusion metrics using QSI analysis in the cervical spinal cords of patients with early cervical spondylosis. Our findings of decreased FA values and increased ADC values are consistent with those of previous studies [6, 7, 23], and they can be explained in part by increased permeability of the membranes due to chronic hypoperfusion [24] and disturbance of the arrangement of axons in the spinal cord [7].

In addition to this increase in ADC values, RMSD values in compressed spinal cords were greater than those in uncompressed cords, meaning that the space for free water was enlarged. In general, RMSD is not influenced by the viscosity of water. Moreover, using the relative maximum high *b* value of 2,000 s/mm^2^ avoids the influence of perfusion of the microcirculation. Therefore, an increase in the RMSD value can be explained instead by an increase in the permeability of the intra- and intercellular spaces.

The decreases in FA and mean DK values can be explained by the possibility that microstructural changes in the spinal cord are independent of the orientation of the fibre tracts [19]. In general, decreased FA values mean axonal damage, i.e. degeneration of the white matter in the brain and spinal cord [25]. However, mean DK values can increase in conditions in which damage occurs along the axon bundles [19]. Therefore, the decrease in mean DK values suggested that the compressed lesions would show structural changes with not only disturbance of the arrangement of axons but also changes in the directions and components of the fibre tracts, including the grey matter. This is a very important finding in terms of evaluation of the cervical spinal cord, because past reports have focused on white matter damage [6, 7, 26]. In fact, when FA values are used as biomarkers for evaluation, the grey matter is difficult to evaluate. Experimental histological studies [27] have shown abnormalities predominantly within the grey matter, and axonal degeneration and obvious demyelination have rarely been seen with compressive cervical myelopathy. This indicates that microcirculatory disturbance plays an important role in the damage in the compressed spinal cord [28]. Therefore, assessment of the cervical spinal cord by using mean DK offers advantages over that using FA because both white matter and grey matter can be better characterized [14, 20, 29].

Overall, this technique may prove to be useful in the assessment of the severity and type of cord abnormality in patients who demonstrate cord narrowing and symptoms without signal change within the cervical cord on conventional imaging. Moreover, this technique may be helpful in demonstrating early cord damage, which may be related to acute or reversible changes (transverse change) or to long-term damage (longitudinal change). In clinical situations, one possible role for this type of imaging evaluation would be to assess changes or progression as part of sequential follow-up of patients where the signs and conventional imaging findings are not sufficient to warrant surgery or invasive therapy but are sufficient to warrant follow-up.

One potential limitation of this study is the relatively low maximum *b* value (*b* = 2,000 s/mm^2^) that we used to calculate RMSD using *q*-space analysis. Here, the effective spatial resolution was the reciprocal of the maximum *q* value, i.e. 25.42 μm. This value seemed to be relatively large for the grey and white matter components, and the RMSD values for the spinal cord may have been inaccurate. However, using higher *b* values (or *q* values) in the clinical setting leads to fatal image degradation. We therefore decided to evaluate the changes in the RMSD values, rather than the absolute values themselves.

Another limitation was the small number of motion probing gradient (MPG) directions. We used 6 directions to reduce the MR data acquisition time. It has been reported that at least 15 different MPG directions are needed to measure mean DK [14]; however, another report has argued that it might be sufficient to measure in only 6 directions to obtain a DK estimate, e.g. in the assessment of multiple sclerosis lesions [30]. We agree with the latter opinion because of the limited MR time available in a busy clinical setting.

A third limitation was the heterogeneous age distribution of the patients. The FA and ADC values of the cervical spinal cord may be influenced by age-related changes. In fact, Mamata et al. [6] reported that 46 % of their patients showed no elevation in ADC of the spinal cord at the narrow spinal canal level, and our results showed no significant changes in ADC values, although they tended to increase with compression. Therefore, longitudinal studies and clinical correlations with the metrics are needed in the future to avoid the influence of age-related changes.

A fourth limitation was that there was no objective assessment concluding that neurological damage was present within the patient groups. Neither a gold standard nor control groups were used in our evaluation. Therefore, a future study investigating the relationship between the diffusion metrics and the symptoms, signs and prognosis in a larger number of patients will be needed.

In conclusion, in addition to FA values, the values of mean DK and RMSD in the spinal cord may reflect microstructural changes and damage with high sensitivity. More studies of the imaging–pathology relationship are needed, but this technique has the potential to provide new information beyond that provided by conventional diffusion-weighted and tensor imaging metrics based on the mono-exponential model.
